# Dietary Supplementation of *Sophora flavescens* Root Extract Improved the Growth Performance, Antioxidant Capacity, Innate Immunity, and Disease Resistance against *Edwardsiella tarda* Challenge in Turbot (*Scophthalmus maximus*)

**DOI:** 10.3390/antiox12010069

**Published:** 2022-12-29

**Authors:** Yuqing Hou, Xuezheng Gao, Xueying Shi, Na Dong, Tongtong Yue, Peiyu Zhang, Haiyan Liu

**Affiliations:** 1 Laboratory of Aquatic Animal Nutrition and Ecology, College of Life Sciences, Hebei Normal University, Shijiazhuang 050024, China; 2 Hebei Key Laboratory of Animal Physiology, Biochemistry and Molecular Biology, Shijiazhuang 050024, China; 3 Hebei Collaborative Innovation Center for Eco-Environment, Shijiazhuang 050024, China

**Keywords:** turbot, *Sophora flavescens* root extract, growth performance, antioxidant capacity, disease resistance

## Abstract

The impacts of dietary supplementation with graded levels of *Sophora flavescens* root extract (SFE) on growth performance, antioxidant capacity, immune status, and resistance against *Edwardsiella tarda* challenge in *Scophthalus maximus* were investigated in this study. In all, 600 turbot (initial body weight: 8.38 ± 0.07 g) were randomly distributed in 12 tanks with 50 fish per tank and fed four experimental diets supplemented with 0, 0.05%, 0.1%, or 0.2% SFE (named as: SFE0, SFE0.05, SFE0.1, and SFE0.2, respectively), for 56 days. The results showed that 0.1% and 0.2% SFE supplementation have significantly increased the FBW, WGR, SGR, and PER of turbot, while decreased the FCR of turbot (*p* < 0.05). Dietary SFE supplementations have significantly increased the activities of plasma SOD, CAT, GPx, T-AOC, GST and LZM, decreased plasma MDA contents in turbot under normal or challenge condition (*p* < 0.05). Meanwhile, SFE addition dramatically enhanced the hepatic mRNA expression of antioxidant parameters (including Nrf2, Keap1, SOD, CAT, Trx2, GST and GR) during the normal condition. mRNA levels of NF-κB p65, IκBα, TNF-α, TGF-β, and IL-10 in the liver of fish were notably up-regulated by SFE treatment during normal condition (*p* < 0.05), while the transcription of IL-1β was down-regulated by SFE whenever under normal or challenge condition. 0.1% and 0.2% SFE administration have significantly increased the survival rate of turbot against *E. tarda* challenge (*p* < 0.05). In conclusion, dietary SFE supplementation improved the growth performance, antioxidant activity and disease resistance of turbot, and SFE could be a potential feed additive for turbot.

## 1. Introduction

Nowadays, aquaculture is faced with great challenges due to intensive culture modes, and fish are more susceptible to oxidative stress and infectious diseases [1]. Turbot, *Scophthalmus maximus* L., is an important industrial mariculture species in Northern China, with an annual output of about 60,000 tons, and has a good culture prospect. However, the high-density intensive culture usually led to disease out-breaking in turbot culture. Antibiotics are used to prevent bacterial pathogens and reduce the economic loss of aquaculture [2]. The indiscriminate use of antibiotics can be hazardous to the environment and aid in the evolution of antimicrobial-resistant genes [3,4,5], which has also become a major problem hindering the sustainable development of turbot aquaculture. Hence, more attention has been drawn to search for safe, practical, and effective immunostimulants or antioxidants to protect and improve the health of fish in the aquaculture industry.

Herbs have been used as immunostimulants in traditional medicine for thousands of years [6]. Many medicinal plants including Cornelian cherry (*Cornus mas* L.), *Bougainvillea glabra* leaf meal, dandelion extract are proved useful in controlling diseases in aquatic organisms and in boosting host immune responses [7,8,9]. It is believed that herbs have a great development prospect to replace the use of antibiotics [10,11]. Herbs contain various bioactive compounds, such as flavonoids, polysaccharides, alkaloids, and volatile oils, which work alone or in combination with other feed additives [12,13]. These bioactive compounds could promote growth performance, immunity, digestive enzyme activities, antioxidant status, disease resistance, and stress management of fish [14,15]. Therefore, using herbs as immunostimulants for aquatic animals might be a promising approach to dealing with disease outbreaks in aquaculture.

*Sophora flavescens* root is a kind of traditional medicinal herbs and has a long history in the traditional medicine of many countries, such as China, Japan, Korea, India, and some countries in Europe. More than 200 compounds are isolated from *S. flavescens* root, the main bioactive components are alkaloids and flavonoids [16]. Previous studies on human medicine demonstrated that *S. flavescens* had a wide range of biological properties, such as anti-inflammatory, antioxidant, antibacterial, antiviral, antitumor, and hepatoprotective actions [17,18]. However, few researches on *S. flavescens* were reported in aquatic animals except for tilapia and flounder. *S. flavescens* could increase the activities of non-specific immune enzymes in tilapia (*Oreochromis niloticus*) and improve the survival rate of tilapia infected with *Streptococcus agalactia* [19]. It can also improve the lysozyme activity and phagocytic activity of flounder (*Paralichthys olivaceus*), and enhance the disease resistance to *Edwardsiella tarda* [20]. There is an urgent need for more researches on *S. flavescens* in aquatic animals.

Nrf2 (nuclear factor erythroid 2-related factor 2) and NF-κB (nuclear factor kappa B) signaling pathways play a major role in the regulation of oxidative stress as well as immune response in aquatic animals [21,22,23]. Nrf2 transcription factor regulates the oxidative stress response and also suppresses the inflammatory response [24], after that the NF-κB immunity regulatory pathway could affect oxidative stress and ROS levels [25]. It is proved that *S. flavescens* had a strong role in improving antioxidant capacity and innate immunity by regulating Nrf2 and NF-κB signal pathway in mammals [26]. *S. flavescens* promoted Nrf2 translocation to the nucleus with subsequently up-regulating antioxidative enzyme protein expression, and then enhanced antioxidant capacity [27]; it could also suppress the phosphorylation of inhibitor of κBα (IκBα), interfere with the translocation of NF-κB from cytoplasm to nucleus, and affect the expression of downstream inflammatory factors, so as to enhance the body immunity of mammals [28]. Therefore, we hypothesized that *S. flavescens* root extract has the potential to enhance the antioxidant capacity and disease resistance of turbot by activating the Nrf2 and NF-κB signaling pathways.

The objective of the current study was to investigate the effects of dietary administration of *S. flavescens* root extract on the growth performance, antioxidant activity, innate immune response, disease resistance, and the expression of genes associated with the Nrf2 and NF-κB signaling pathways.

## 2. Materials and Methods

### 2.1. Experimental Diets

*Sophora flavescens* root extract (SFE) was purchased from Xi’an Shengqing Biological Technology Co. Ltd. (Xian, China), and it is the ethanolic extract of *S. flavescens* root. (Including: 97.35 mg matrine/kg and: 97.82 mg oxymatrine/kg, determined by HPLC). Four iso-nitrogenous and iso-energetic experimental diets with 0, 0.05%, 0.1% or 0.2% SFE (named as: SFE0, SFE0.05, SFE0.1, and SFE0.2, respectively), were prepared. The experimental diets used white fish meal and chicken meal as primary protein sources, fish oil as chief lipid sources, α-starch as the main carbohydrate source.

The feed ingredients were mixed and ground sufficiently through a 178 μm mesh sieve. Then, the fish oil was added to the powder and mix the ingredients. All ingredients were supplemented the distilled water and mixed adequately to form into 2 mm pellets by extruded pelletizer (EL-260, Youyi Machinery Factory, Weihai, Shandong, China), which were placed in a ventilated place to dry at room temperature for 30 min. The prepared diets were stored at −20 °C for further use. The dietary formulation and proximate compositions are shown in Table 1.

### 2.2. Fish and Growth Trial

Turbots were obtained from a commercial aqua-farm (Tianjin, China). All fish are acclimated to the laboratory conditions and fed the prepared basal diet for 2 weeks. After acclimatization period, 600 fish (average weight: 8.38 ± 0.07 g) were assigned to twelve tanks (capacity: 560 L) with three replicates per treatment and 50 fish per tank. Fish were hand-fed the corresponding diets twice a day (8:00 and 18:00) to apparent satiation. The feeding trial was conducted for 56 days. During the feeding trial, water temperature was maintained at 16–18 °C, salinity at 15–20‰, ammonia nitrogen lower than 0.05 mg/L and dissolved oxygen higher than 6.0 mg/L.

### 2.3. Sample Collection

At the end of feeding trial, all fish were anesthetized with 100 mg/L MS-222 solutions (Sigma-Aldrich, St. Louis, MO, USA) and batch weighed. Four fish per tank were dissected and stripped to calculate VSI and HSI. Two fish per tank were used for chemical analysis and gene expression. The blood of fish was collected from the tail vein of the syringe moistened by heparin sodium and centrifuged with 3000× *g* 15 min to measure the plasma non-specific immune and antioxidant indexes. The liver was stripped to analyze the mRNA expression. All the samples were immediately frozen in liquid nitrogen and stored in the refrigerator at −80 °C until use.

After feeding trial, 20 fish per tank were exposed to bacteria challenge test. *Edwardsiella tarda* was provided from Fish Disease Laboratory of Hebei Agricultural University. The bacteria were inoculated into TSA solid medium and cultured at 30 °C for 12 h. Then the single bacteria were selected in the aseptic beef soup liquid medium, cultured in 200 rpm shaker at 30 °C for 24 h, 4000 rpm centrifugation 10 min, remove the supernatant, rinse with 3.5% aseptic salt water and dilute the precipitated bacteria. The fish in 12 tanks were injected intraperitoneally with 3 × 10^8^ CFU/mL *E. tarda* solution 0.3 mL (the injection concentration and dose were determined by the pre-experiment). Mortality was observed for 7 days, the dead fish was counted every 6 h and the survival rate was calculated. The plasma and liver of turbot were sampled for further analysis after 72 h challenge as the process described above.

### 2.4. Chemical Analysis

The proximate compositions of diets were analyzed according to standard methods, as previously described [29]. Moisture was determined by oven drying at 105 °C to constant weight. Crude protein (N × 6.25) was measured by Kjeldahl method using a 4800 Kjeltec Analyzer Unit (FOSS Tecator, Haganas, Sweden). Crude lipids were detected by petroleum ether extraction using the Soxhlet method. Crude ash was determined by combustion in a muffle furnace at 550 °C for 12 h. Gross energy was evaluated by an oxygen calorimeter (Parr 6300, Parr Instrument Company, Houston, TX, USA).

Plasma parameters, including lysozyme (LZM), complement 3 (C3), immunoglobulin (IgM), catalase (CAT), superoxide dismutase (SOD), glutathione peroxidase (GPx), total antioxidant capacity (T-AOC), glutathione-S-transferase (GST) activities, glutathione reductase (GR) and malondialdehyde (MDA)content were measured using commercial kits (Jiancheng Bioengineering Ltd., Nanjing, China). The determination method and calculation follow its instructions. The LZM, complement 3 and IgM activity were measured by turbidimetric assay. The activity of CAT was measured according to visible light spectrophotometry. The activity of SOD activity was evaluated according to hydroxylamine method. The T-AOC present in plasma was measured according to ABTS method. The MDA activity in the plasma was measured by means of a TBA method. These parameters were analyzed using a microplate reader (BioTek Instruments, Inc., Winooski, VT, USA) to determine the contents. Activities of GPx and GR were determined by recording the colorimetry through TU-1810 spectrophotometer (Beijing Purkinje General Instrument Co., Ltd., Beijing, China).

### 2.5. Real-Time Quantitative PCR Analysis

Total RNA of liver was extracted with Trizol reagent (Shanghai Generay, Shanghai, China). The RNA concentration and purity were evaluated with a Nano-Drop ND-2000 spectrophotometer (Thermo Scientific, Wilmington, DE, USA). The quality of RNA was assessed by 1.2% (*w/v*) agarose gel electrophoresis. Complementary DNA (cDNA) was synthesised using the cDNA synthesis kit (RevertAid First Strand cDNA Synthesis Kit, Thermo Scientific, Wilmington, DE, USA). Ribosomal protein S4 (RPS4) was applied as housekeeping gene, Real-time quantitative PCR analyses were performed using the CFX96 Real-Time PCR system (Bio-Rad, Hercules, USA) following standard protocols. Reaction mixtures of 20 μL (0.4 μL forward primer, 0.4 μL reverse primer (10 μM), 10μL 2× TransStart^®^ Top Green qPCR SuperMix (TransGen Biotech, Beijing, China), 2 μL cDNA (200 ng/μL) and 7.2 μL RNase-free water) were amplified for 30 s at 94 °C followed by 45 cycles of 5 s at 94 °C, annealing for 30 s, finally, 30 s at 72 °C. The primers used for qRT-PCR were listed in Table 2. The amplification efficiencies of all primers were verified to be approximately 100%. The relative expression levels of the target gene were calculated by 2^−ΔΔCt^ methods [30]. Six samples were employed for each treatment and each sample was tested in duplicate.

### 2.6. Statistical Analysis

All data were presented as mean values and standard deviation (S.D.). Statistical analyses were performed using the STATISTICA 10.0 (StatSoft Inc., Tulsa, OK, USA) software, the normality and variance homogeneity of data were tested. The parameters of growth (including FBW, FR, WGR, SGR, FCR, PER, SR) and morphology (containing CF, VSI, HSI) were analyzed by one-way ANOVA. Two-way ANOVA was employed in plasma biochemistry indexes (including LZM, Complement C3, IgM, CAT, SOD, T-AOC, GPx, GST, GR, and MDA) and gene expression parameters. Duncan multiple comparisons were used when there was a significant difference in ANOVA analysis, and *p* < 0.05 was considered statistically significant.

## 3. Results

### 3.1. Growth Performance

The effects of *S. flavescens* root extract (SFE) on the growth performance and morphometric parameters of turbot are presented in Table 3. No significant differences were observed in SR, FR, VSI and HSI (*p* > 0.05). The FBW, WGR, SGR and PER in SFE 0.1 and SFE 0.2 treatments were significantly higher than that in SEF 0 group (*p* < 0.05). Dietary SFE supplementation significantly reduced the FCR values in SFE 0.1 and SFE 0.2 groups (*p* < 0.05), the lowest value was at SFE 0.1 group. CF was significantly highest in the SFE 0.1 group compared to other groups (*p* < 0.05).

### 3.2. Plasma Antioxidant and Lipid Peroxidation Parameters

Table 4 showed the effects of dietary SFE supplementation on the plasma antioxidant parameters. SFE supplementations have significantly increased the activities of plasma SOD, CAT, GPx, T-AOC, and GST of turbot under normal or challenge condition (*p* < 0.05). The highest levels existed in SFE 0.1 group except for CAT and GPx under challenge condition, which were highest in SFE 0.05 group. The plasma MDA contents were dramatically decreased by SFE addition, and the lowest concentration was in SFE 0.1 treatment under normal and challenge condition (*p* < 0.05). No significant effect of SFE on plasma GR activities were observed in this study (*p* > 0.05).

### 3.3. Plasma Immune Indexes

Dietary supplementation with SFE positively influenced the plasma LZM (*p* < 0.05). The LZM was highest in the SFE 0.1 group compared with that in other groups. However, no significant difference was observed in plasma complement C3 and IgM levels among all groups (*p* > 0.05) (Table 5).

### 3.4. Antioxidant-Related Gene Expression

**Figure 1:**
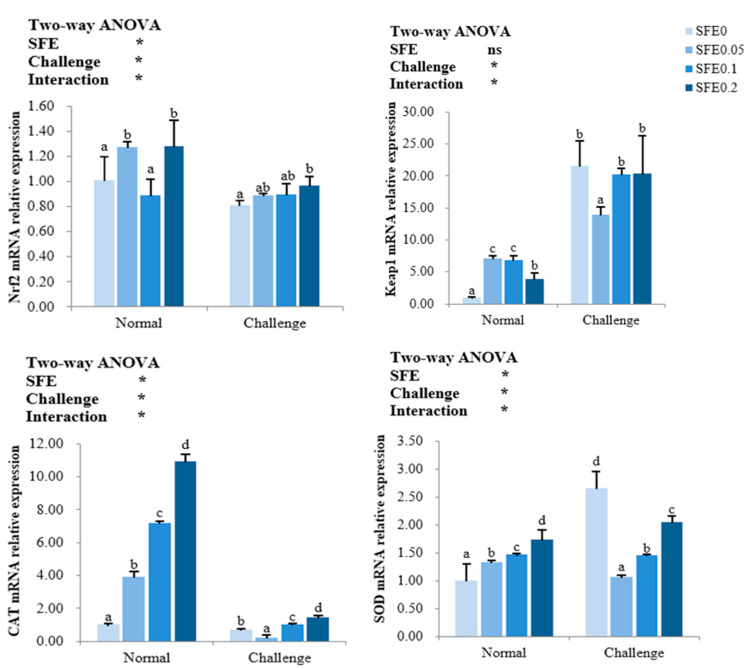
This relative transcription of nuclear factor erythroid 2-related factor 2 (Nrf2), kelch-like ECH-associated protein 1 (Keap1), catalase (CAT), superoxide dismutase (SOD) of turbot fed varied levels of SFE for 8 weeks. Normal refers to the parameters of the breeding group, and Challenge refers to the parameters of the challenge group. Values represent the mean ± S.D. (*n* = 6). Different letters indicate a significant difference between the groups fed different SFE diets (*p* < 0.05). ns: *p* > 0.05, *: *p* < 0.05.

As seen from Figure 1, SFE treatment has significantly affected the Nrf2 signaling pathway and its downstream genes expression. In the normal condition, SFE has dramatically enhanced the mRNA levels of Nrf2 and Keap1 expect for Nrf2 in SFE 0.1 group (*p* < 0.05), the Nrf2 gene expression increased with the increasing of dietary SFE levels after pathogen challenge, it is significantly higher in SFE 0.2 than in SFE 0 group (*p* < 0.05). The SFE additions have significantly increased the mRNA levels of CAT and SOD during the normal condition. When exposed to pathogen challenge, the gene expressions of CAT, SOD in SFE 0.05 group were significantly lower than that in other treatments (*p* < 0.05).

**Figure 2:**
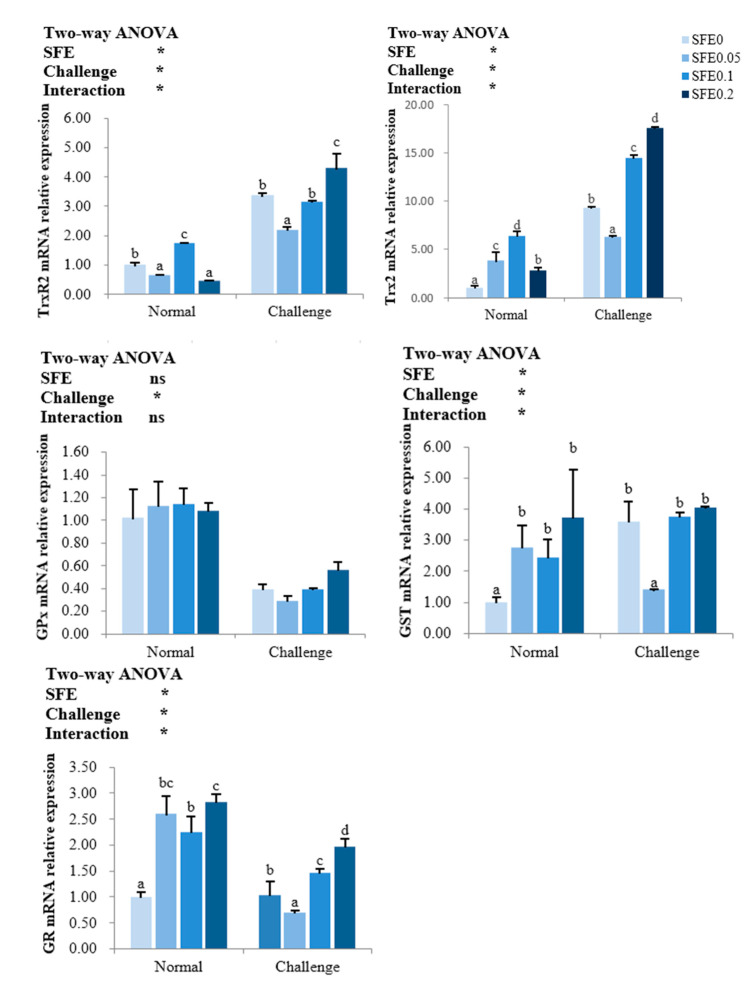
Relative transcription of thioredoxin reductase 2 (TrxR2), thioredoxin 2 (Trx2), glutathione peroxidase (GPx), glutathione S-transferase (GST), glutathione reductase (GR) of turbot fed varied levels of SFE for 8 weeks. Normal refers to the parameters of the breeding group, and Challenge refers to the parameters of the challenge group. Values represent the mean ± S.D. (*n* = 6). Different letters indicate a significant difference between the groups fed different SFE diets (*p* < 0.05). ns: *p* > 0.05, *: *p* < 0.05.

The transcription levels of the thioredoxin regulatory system and glutathione antioxidant regulatory system related genes are presented in Figure 2. The gene expression of GPx was not affected by SFE treatment (*p* > 0.05), while SFE additions have significantly increased the mRNA levels of Trx2, GST and GR during the normal condition, the highest transcription levels of Trx2 and TrxR2 was in SFE 0.1 group, the highest mRNA expressions of GST and GR were observed in SFE 0.2 group. When exposed to pathogen challenge, the gene expressions of TrxR2, Trx2, GST and GR in SFE 0.05 group were significantly lower than that in other treatments (*p* < 0.05).

### 3.5. Inflammation-Related Gene Expression

**Figure 3:**
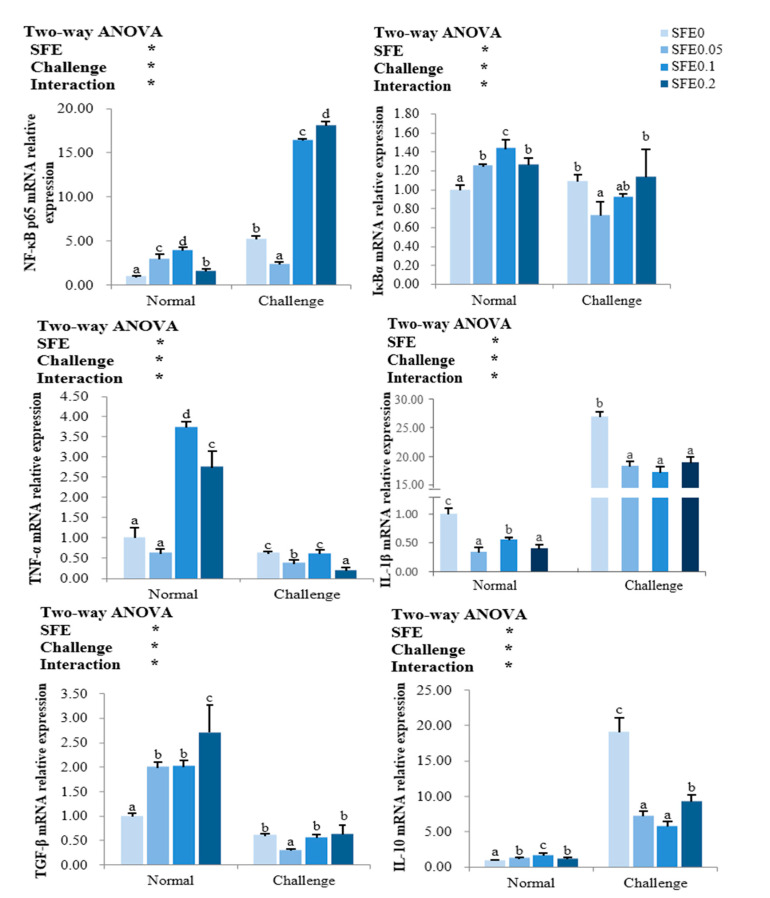
Relative transcription of nuclear factor kappa B–p65 (NF-κB p65), inhibitor of κBα (IκBα), tumor necrosis factor-alpha (TNF-α), interleukin-1 beta (IL-1β), transforming growth factor beta (TGF-β), interleukin -10 (IL-10) of turbot fed varied levels of SFE for 8 weeks. Normal refers to the parameters of the breeding group, and Challenge refers to the parameters of the challenge group. Values represent the mean ± S.D. (*n* = 6). Different letters indicate a significant difference between the groups fed different SFE diets (*p* < 0.05). ns: *p* > 0.05, *: *p* < 0.05.

SFE treatment significantly regulated the key regulators NF-κB p65 and IκBα in the NF-κB signaling pathway, and their downstream pro-inflammatory factors TNF-α and IL-1β, anti-inflammatory factors TGF-β and IL-10 (*p* < 0.05) (Figure 3). Under normal condition, mRNA levels of NF-κB p65, IκBα, TNF-α, TGF-β and IL-10 in the liver of fish were notably increased by SFE treatment (*p* < 0.05), NF-κB p65, IκBα, TNF-α, and IL-10 increased first and then decreased, and reached the highest value in the SFE 0.1 group (*p* < 0.05). Anti-inflammatory cytokine TGF-β expression was the highest in SFE 0.2 group. Dietary SFE significantly decreased the transcription levels of pro-inflammatory cytokines IL-1β (*p* > 0.05) whenever under normal or challenge condition. After pathogen challenge, the mRNA levels of NF-κB p65 were significantly increased in SFE 0.1 and SFE 0.2 groups. Pro-inflammatory cytokines TNF-α was down-regulated in the SFE 0.05 and SFE 0.2 groups. Anti-inflammatory cytokine IL-10 were down-regulated in all SFE treatment groups (*p* < 0.05).

### 3.6. Disease Resistance

**Figure 4:**
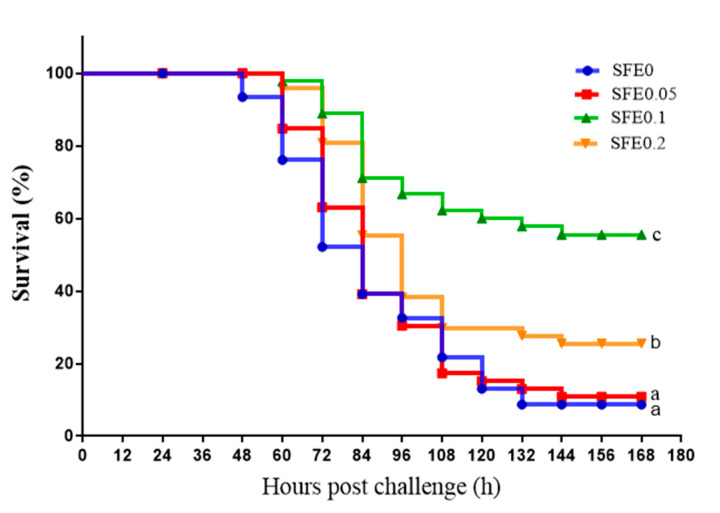
The survival rate of turbot fed varied levels of SFE when exposed to *Edwardsiella tarda* challenge for 7 days. Values are presented as means ± S.D. Different letters indicate significant differences among all treatments (*p* < 0.05).

After pathogen challenge, dead of turbot was observed firstly in SFE 0 group, and then the mortality was dramatically increased during 2–6 days post challenge. Dietary supplementation with SFE increased the survival rate of turbot exposed to pathogen challenge (Figure 4). The survival rates in SFE 0 and SFE 0.05 were significantly lower than that in SFE 0.1 and SFE 0.2 groups (*p* < 0.05).

## 4. Discussion

Many herbs or their compounds have been used as supplements and additive in animals as well as human diets because of their numerous beneficial properties such as growth promotion, anti-cancer, anti-pathogenic, anti-inflammatory, immunomodulatory, and antioxidant properties [13]. In recent years, there has been increasing interest in using natural plant extract as functional compounds to enhance the immunity of fish in aquaculture. The root extracts of *S. flavescens*, a kind of traditional Chinese herbal medicine, has a wide range of medicinal values, such as antibacterial and anti-inflammatory [16]. However, little information was available about the functions and regulating mechanism of *S. flavescens* in fish. This study demonstrated that *S. flavescens* root extract enhanced the growth performance, antioxidant capacity, immunity, and disease resistance of turbot.

### 4.1. SFE improved the Growth Performance and Feed Utilization of Fish

In this study, 0.1% and 0.2% dietary SFE supplementation significantly increased the growth performance and feed utilization of fish, which is similar with previous studies of plant herbs extract. It is reported that *Radix glycyrrhizae* extract, *Panaxnotoginseng* extract and tea tree oil have increased the WGR, SGR of yellow catfish (*Pelteobagrus fulvidraco*), grouper (*Epinephelussp*) and prawn (*Macrobrachium rosenbergii*) [31,32,33], respectively. This is probably related to the bioactive compounds isolated from these herbs. It is proved that the bioactive substances contained in Chinese herbal medicine are the main factors to promote the rapid growth of aquatic animals [15]. Many studies have shown that alkaloids (such as berberine), flavonoids, triterpenes (such as hesperidin) and polysaccharides (such as Astragalus polysacharin) extracted from Chinese herbal medicine can significantly increase fish FBW, WGR and SGR, and reduce FCR [34,35,36,37]. The composition of *S. flavescens* extract is complex, more than 200 compounds are isolated from *S. flavescens* root, and the main bioactive components are alkaloids and flavonoids [16]. It might be ascribed to these bioactive components from SFE improved the antioxidant capacity and non-specific immune responses, which in turn boosts turbot growth performance.

### 4.2. SFE Enhanced the Antioxidant Capacity of Fish

Antioxidant enzymes are indicative biomarkers of health and the reaction in oxidative stress response when the reactive oxygen species (ROS) and free radicals production were uncontrolled [38], and MDA is regarded as a bio-indicator of oxidative stress [39]. In the current study, the activities of plasma CAT, SOD, T-AOC, GPx, GST, and GR were significantly increased, and plasma MDA contents were decreased in fish fed *S. flavescens* diets. The results of this study indicated that *S. flavescens* extract could improve the antioxidant capacity of juvenile turbot. Previous research has confirmed that *S. flavescens* can trigger the antioxidant defense system of mammals [40]. Similarly, antioxidant enzymes activity increased significantly in fish with dietary administration of grape seed proanthocyanidin extract [41] or curcumin [42], and decreased the MDA levels in the fish. The current results proved that *S. flavescens* extract improved the antioxidant system of turbot and reduced oxidative stress. This may be because the bioactive components in *S. flavescens* extract can activate the antioxidant related pathway Nrf2 and thus lead to the change of enzyme activity level. As we know, the increased enzyme activity is caused by the increased synthesis of enzyme protein, which largely relies on its gene transcription and translation [43]. Therefore, in order to further confirm whether the change of antioxidant enzyme activity is caused by the change of mRNA level of related genes in Nrf2 signaling pathway, this study determined the influence of *S. flavescens* extract on the expression level of antioxidation-related genes.

A number of reports have demonstrated the importance of the Nrf2- Keap1 signaling pathway against oxidative stress in the body. The nuclear transcription factor Nrf2 is widely expressed in an extensive range of cell and tissue types and regulates antioxidant defense [44,45]. Nrf2 transcriptional activity can be inhibited by Keap1-controlled ubiquitination-proteasomal degradation [46]. Nrf2 translocates into the nucleus and binds ARE to regulate and code are involved in redox regulation (CAT, SOD and Trx), and enzymes related to glutathione synthesis metabolism, such as GST, GR, GPx, etc., to regulate oxidation in the body [47]. In this study, *S. flavescens* extract supplementation activated Nrf2/Keap1 signaling pathway under normal condition, thus increased the expression of downstream related antioxidant genes, the mRNA expression results were consistent with the results of their enzyme activities under normal condition, which revealed that dietary SFE could strengthen antioxidant activity associated with decreased MDA content of turbot. Similar to our research, dietary supplementations of *P. tectorius* in *C. carpio* [48], emodin in *M. amblycephala* [49], curcumin in *C. argus* [42] regulated the expression of Nrf2, Keap1 and its downstream regulated genes such as SOD, CAT, GPx and GST to improve the antioxidant activity in fish. To date, no reports have investigated the potential effects of *S. flavescens* on the thioredoxin regulatory system of fish. The thioredoxin system is one of the major redox systems in cells and is an important regulator of eliminating ROS accumulation, the main components are thioredoxin (Trx) and thioredoxin reductase (TrxR) [50,51]. In the current study, the expressions of hepatic Trx2 and TrxR2 were significantly up-regulated in turbot fed an *S. flavescens* extract diet. *S. flavescens* extract increases the expression of thioredoxin and its related reductases by activating the Nrf2/Keap1 signaling pathway, and further improves the antioxidant capacity of turbot, so that it can quickly eliminate excessive ROS to protect the body. Furthermore, it is interestingly found in this study that the expression of SOD was down-regulated in SFE groups compared to control after 72 h pathogen challenge. There probably exists the negative feedback suppression. The activities and mRNA expression of SOD in SFE treatments were all at higher levels during normal condition, which showed there are enough SOD activities in SFE treatments to defense the bacterial invasion to maintain the body homeostasis, no need to activate the genes to produce more enzymes. Hence, in this study, the transcriptional levels of SOD in SFE groups were not significantly increased by *E. tarda* challenge, while the level in SFE 0 significantly increased, which revealed that dietary SFE supplementation could protect turbot from pathogen persecution. Based on the results of antioxidant parameters determined in this study, a dietary supplement of SFE improved the antioxidant capacity of turbot by activating the Nrf2 pathway and enhancing antioxidant systems including SOD, CAT, GPx, GST and Trx and protected lipids from peroxidation.

### 4.3. SFE Enhanced the Innate Immunity and Disease Resistance of Fish

Lysozyme, the first line of protection against pathogen invasion, plays a key role in non-specific immune response in fish. It can cleave bacteria by hydrolyzing the β-1,4 glycosidic bond of peptidoglycan layer of the bacterial cell wall [52]. In this study, *S. flavescens* exerted its antibacterial effect by increasing the activity of plasma LZM and enhancing immunity in juvenile turbot. This is consistent with the results in tilapia and flounder [19,20]. Similarly, dietary *Eucommia ulmoides* Oliver and Astragalus polysaccharides increased LZM in turbot [37,53]. *Allium mongolicum* Regel significantly improved LZM in *Channa argus* [35]. This indicated that the bioactive substances in the extracts of Chinese herbal medicine could improve the activity of LZM, and then improve the immunity of fish.

Moreover, the transcription levels of immune-related genes were analyzed in this study to further evaluate the effects of SFE on immune responses of turbot. NF-κB pathway is involved in the initiation, amplification and regression of inflammation and is activated by different stimuli [54,55]. Cytokines play a vital role in the immune system by binding to specific receptors and setting off a cascade of immunological events [56]. Pro-inflammatory cytokines (IL-1β, TNF-α) regulate multiple aspects of the immune response in fish [57]. TGF-β and IL-10, anti-inflammatory cytokine, suppresses the production of pro-inflammatory cytokines [58]. In this study, SFE treatment activated the expression of NF-κB p65 and IκBα in the normal condition, then up-regulated the mRNA expression of anti-inflammatory cytokine (TGF-β and IL-10), and down-regulated the mRNA expression of pro-inflammatory cytokines (IL-1β). Similar results were found in other plant extract studies, such as turmeric in *Cyprinus carpio* [59], *Allium mongolicum Regel* in *Channa argus* [35]. However, in the present study, the transcription of TNF-α, another anti-inflammatory cytokine, were up-regulated sharply in SFE 0.1 and SFE 0.2 groups. TNF-α is a pro-inflammatory cytokine secreted from activated macrophages, vital in regulating innate immune functions and inflammatory responses [60]. In previous studies, dietary *P. tectorius* extract and *Agaricus bisporus* polysaccharides up-regulated TNF-α expression in the fish [61,62], while dietary turmeric significantly decreased the expression of TNF-α in *Cyprinus carpio* [59]. The difference of gene expression results may be due to the difference of the type and dose of plant immune stimulants, feeding time and fish species. Under challenge condition, the gene expressions of NF- κB p65 and IκBα in SFE0 group were up-regulated compared with normal condition, and the mRNA levels of IL-1β and IL-10 increased sharply, which means that excessive inflammatory response occurred in turbot after *E. tarda* infection. Meanwhile, dietary SFE administration significantly decreased the mRNA levels of IL-1β and IL-10, which suggested that dietary supplementation of SFE could inhibit the inflammatory response of turbot infected by *E. tarda* to protect the body from excessive inflammatory reaction.

Due to the prevalence of industrialized intensive farming, fish usually face greater risks of antioxidant stress and pathogen attack. *E. tarda*, a causative infectious agent of disease in aquaculture, caused mass mortalities and considerable economic losses, particularly in turbot [63]. Bacterial challenge test is often used as an effective indicator of fish immunity and health status. In this study, after the challenge, turbot in SFE 0 treatment firstly displayed the clinical symptoms of *E. tarda* infection: red bleeding plaques in the head and muscles, dark red mucus in the gills, blood spots on the fins, anal congestion, and protrusion. Then, the first dead turbot was observed in the SFE 0 group on the second day, the mortality lasted to the sixth day after challenge. Further, 0.1% SFE administration significantly relieved the symptoms and increased the survival rate of turbot. The survival rates of SFE administration groups were higher than that of SFE 0 group. Therefore, the elevation of disease resistance against *E. tarda* is contributed to the regulation of immune response and antioxidant capacity by SFE administration. That is to say, SFE could affect mRNA levels of immune-related genes by activating Nrf2/Keap 1 and NF-κB signaling pathways, and then improved the antioxidant capacity and immunity of turbot, which also improved the ability of juvenile fish to resist *E. tarda* infection. It also suggests that antioxidant capacity, immunity, and disease resistance are inextricably linked.

## 5. Conclusions

**Figure 5:**
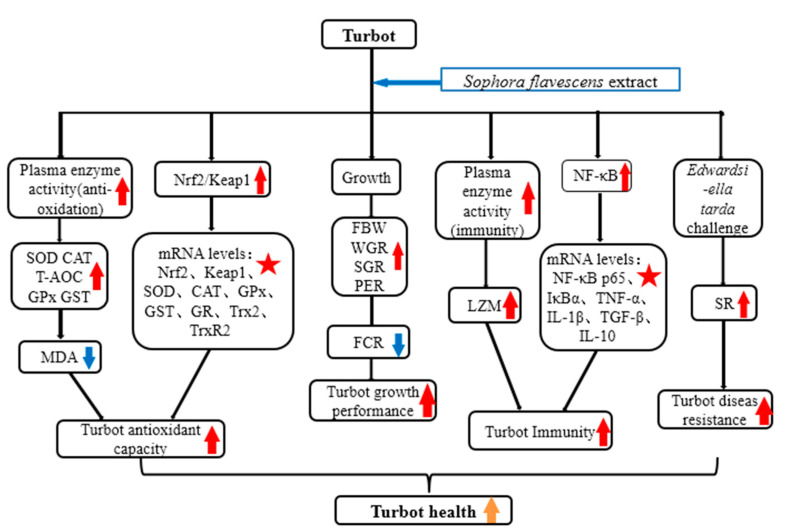
General summary for the beneficial effects and potential mechanisms of SFE on the health of turbot. (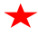: indicates a significant impact. The red arrow indicates the promoting effect, the blue arrow indicates the inhibitory effect, and the orange arrow indicates a positive effect on fish health).

Overall (Figure 5), the present study was apparently the first report that dietary SFE supplementation for 56 days promoted the immunity and antioxidant capacity through activating Nrf2/Keap1 and NF-κB signal pathways, thus improving the growth performance and disease resistance against *E. tarda*. SFE could be a potential feed additive for turbot.

## Figures and Tables

**Table 1:** Formulation and nutrient composition of experimental diets (dry weight basis, %).

Ingredients	SFE 0	SFE 0.05	SFE 0.1	SFE 0.2
White fish meal	40.00	40.00	40.00	40.00
Chicken meal	17.50	17.50	17.50	17.50
Dried squid liver meal	5.00	5.00	5.00	5.00
Wheat gluten	5.00	5.00	5.00	5.00
Yeast powder	4.00	4.00	4.00	4.00
α-starch	18.00	17.95	17.90	17.80
Fish oil	4.00	4.00	4.00	4.00
*Sophora flavescens* root extract	0.00	0.05	0.10	0.20
CaHPO4	3.00	3.00	3.00	3.00
Zeolite powder	1.30	1.30	1.30	1.30
Choline chloride	0.20	0.20	0.20	0.20
Premix	2.00	2.00	2.00	2.00
Nutrient composition				
Crude protein	48.61	48.54	48.52	48.58
Crude lipid	10.53	10.54	10.56	10.51
Crude ash	16.58	16.60	16.62	16.68
Gross energy (MJ/kg)	19.54	19.59	19.60	19.56

Premix: According to Zhang et al. (2023) [29].

**Table 2:** Primer sequences for RT- qPCR.

Target Gene	Primer Sequence	Annealing Temperature (°C)	Product Size (bp)	Accession Number
NF-κB p65	F:ACTCACCCAGCCATCAAG R:AAGCAAAGCCGAACTGAA	58	314	XM_035627239.1
IκBα	F:AGAAAGCAGGAAATCAACTAAG R:TTGCGACTGACGATAAGG	58	205	XM_035649251.1
TNF-α	F:TGGAGATGGGTCTTGAGG R:TGGCATTGCTGCTGATTT	55	249	XM_035629860.2
IL-1β	F:ATGGTGCGATTTCTGTTCTAC R:TTCCACTTTGGGTCGTCTT	50	204	XM_035640817.2
TGF-β	F:TCAGCATTCCAGATGTAGGTG R:GGAGAGTGGCTTCAGTTTTTC	54	312	XM_035623668.2
IL-10	F:CCACGCCATGAACAGCATCCT R:ACATCGGACTTGAGCTCGTCGAA	60	141	XM_035632547.1
Nrf2	F:GGCAAGAACAAAGTGGCT R:GCAGACGCTCTTTCTCATC	59	106	XM_035651303.1
Keap1	F:TTGCCGAGCAGATTGGTT R:AGCGGACAGCCTGGAGTA	58	249	XM_035636756.1
Trx2	F:GGCTCACAGGCTGCTCGTA R:GGGCAGTTCGCTGTTGAT	60	253	XM_035616077.1
TrxR2	F:GAGGCTGTTCACAACCACG R:CCACCTGAGGCGATTACG	60	182	XM_035640386.1
GPx	F:CCCTGATGACTGACCCAAAG R:GCACAAGGCTGAGGAGTTTC	57	174	XM_035632618.1
GR	F:GTCTCCTCTGGGCTATTGG R:CGTGATACATCGGAGTGAAA	55	375	XM_035650249
GST	F:TGGATTACTTCACTGGACCTT R:TTTACCTATGAGTCGTCGTTC	53	272	XM_035636617
SOD	F:AAACAATCTGCCAAACCTCTG R:CAGGAGAACAGTAAAGCATGG	58	165	MG253620.1
CAT	F:TCCCGTCCTTCATTCACT R:AATAGCATAATCTGGGTTGGT	57	205	MG253621.1
RPS4	F:CAACATCTTCGTCATCGGCAAGG R:ATTGAACCAGCCTCAGTGTTTAGC	60	143	XM_035608277.1

NF-κB p65, nuclear factor kappa B–p65; IκBα, inhibitor of κBα; TNF-α, tumor necrosis factor-alpha; IL-1β, interleukin-1 beta; TGF-β, transforming growth factor beta; IL-10, interleukin -10; Nrf2, nuclear factor erythroid 2-related factor 2; Keap1, kelch-like ECH-associated protein 1; Trx2, thioredoxin 2; TrxR2, thioredoxin reductase 2; GPx, glutathione peroxidase; GR, glutathione reductase; GST, glutathione S-transferase; SOD, superoxide dismutase; CAT, catalase; RPS4, ribosomal protein S4.

**Table 3:** Effects of dietary SFE on growth performance and morphometric parameters of turbot.

Indices	SFE0	SFE0.05	SFE0.1	SFE0.2	*p* Value
IBW (g)	8.40 ± 0.10	8.40 ± 0.00	8.37 ± 0.06	8.37 ± 0.12	0.916
FBW (g)	25.31 ± 1.03 ^a^	26.45 ± 0.42 ^ab^	30.00 ± 0.90 ^c^	27.67 ± 0.87 ^b^	<0.001
FR (%/d)	1.38 ± 0.06	1.39 ± 0.04	1.36 ± 0.04	1.32 ± 0.04	0.310
WGR (%)	201.41 ± 15.74 ^a^	214.83 ± 5.01 ^ab^	258.60 ± 9.26 ^c^	230.65 ± 7.83 ^b^	<0.001
SGR (%/d)	1.97 ± 0.09 ^a^	2.05 ± 0.03 ^ab^	2.28 ± 0.05 ^c^	2.14 ± 0.04 ^b^	0.001
FCR	0.77 ± 0.02 ^b^	0.75 ± 0.03 ^b^	0.68 ± 0.01 ^a^	0.69 ± 0.03 ^a^	0.002
PER	2.66 ± 0.09 ^a^	2.73 ± 0.09 ^a^	3.00 ± 0.06 ^b^	2.99 ± 0.11 ^b^	0.003
SR (%)	99.33 ± 1.15	99.33 ± 1.15	98.67 ± 1.15	100.00 ± 0.00	0.487
CF (%)	3.15 ± 0.23 ^a^	3.19 ± 0.24 ^a^	3.41 ± 0.23 ^b^	3.21 ± 0.21 ^a^	0.040
VSI (%)	6.83 ± 0.52	6.78 ± 0.31	6.74 ± 0.44	6.67 ± 0.31	0.814
HSI (%)	2.55 ± 0.43	2.66 ± 0.50	2.64 ± 0.49	2.42 ± 0.43	0.593

Values (mean ± S.D.) in the same line with different superscript letters were significantly different from each other (*p* < 0.05). IBW, initial body weight; FBW, final body weight; Weight gain rate (WGR,%) = ((FBW–IBW)/IBW) × 100; Specific growth rate (SGR, %/d) = ((LnFBW–LnIBW)/days) × 100; Feeding rate (FR, %/d) = 100 × feed intake/(days× (FBW+IBW)/2); Feed conversion ratio (FCR) = (total dry feed intake (g) /wet weight gain (g)); Survival rate (SR, %) = (Final number of fish/Initial number of fish) × 100; Protein efficiency ratio (PER, %) = wet weight gain (g)/protein intake (g); Hepatosomatic index (HSI, %) = (liver weight (g)/body weight (g) × 100; Viscerosomatic index (VSI, %) = (visceral weight (g)/body weight (g) × 100; Condition factor (CF) = 100 × (body weight (g)/body length (cm)^3^).

**Table 4:** Effects of dietary SFE on plasma antioxidant indices of turbot.

Index		Group				Two-Way ANOVA (*p* Value)		
		SFE0	SFE0.05	SFE0.1	SFE0.2	SFE	Challenge	SFE × Challenge
SOD(U/mL)	Normal	59.58 ± 1.94 ^a^	60.13 ± 1.95 ^a^	67.63 ± 1.32 ^c^	63.97 ± 3.62 ^b^	<0.001	<0.001	0.002
	Challenge	73.69 ± 7.01 ^ab^	77.79 ± 1.21 ^bc^	80.14 ± 2.30 ^c^	72.50 ± 1.24 ^a^			
CAT(U/mL)	Normal	5.13 ± 1.16 ^a^	6.07 ± 1.45 ^ab^	7.14 ± 1.31 ^b^	6.55 ± 1.07 ^b^	<0.001	<0.001	<0.001
	Challenge	10.96 ± 4.19 ^a^	30.56 ± 11.73 ^b^	14.32 ± 5.17 ^a^	17.29 ± 4.05 ^a^			
GPx (U/mL)	Normal	30.76 ± 6.21 ^a^	42.72 ± 6.43 ^b^	45.90 ± 12.94^b^	41.18 ± 11.88 ^b^	<0.001	<0.001	<0.001
	Challenge	240.67 ± 20.00 ^a^	331.33 ± 11.60 ^b^	243.33 ± 11.18 ^a^	247.33 ± 27.05 ^a^			
T-AOC (mM)	Normal	0.93 ± 0.08 ^a^	0.98 ± 0.08 ^ab^	1.06 ± 0.05 ^c^	1.00 ± 0.05 ^b^	<0.001	0.022	0.036
	Challenge	0.87 ± 0.01 ^a^	0.92 ± 0.02 ^b^	1.05 ± 0.05 ^c^	1.03 ± 0.03 ^c^			
GST (U/mL)	Normal	17.82 ± 5.14 ^a^	24.43 ± 1.66 ^a^	40.96 ± 16.82 ^b^	17.96 ± 2.75 ^a^	<0.001	0.001	0.104
	Challenge	63.06 ± 5.88 ^a^	51.53 ± 21.79 ^a^	95.29 ± 3.55 ^b^	46.59 ± 17.92 ^a^			
GR (U/L)	Normal	30.55 ± 7.01	35.37 ± 9.78	20.90 ± 12.56	28.62 ± 13.90	0.347	0.012	0.651
	Challenge	21.57 ± 6.71	20.19 ± 10.28	17.68 ± 8.57	19.65 ± 8.12			
MDA (nmol/mL)	Normal	30.38 ± 1.32 ^c^	23.96 ± 1.49 ^b^	19.68 ± 0.88 ^a^	19.68 ± 0.74 ^a^	<0.001	<0.001	<0.001
	Challenge	32.37 ± 6.73 ^b^	26.31 ± 2.71 ^a^	25.43 ± 3.84 ^a^	34.89 ± 4.40 ^b^			

Values (mean ± S.D.) (*n* = 6) in the same line with different superscript letters were significantly different from each other (*p* < 0.05). SOD, superoxide dismutase; CAT, catalase; GPx, glutathione peroxidase; T-AOC, total antioxidant capacity; GST, glutathione S-transferase; GR, glutathione reductase; MDA, malondialdehyde.

**Table 5:** Effects of SFE supplementation on plasma nonspecific immune indices of turbot.

Index	SFE0	SFE0.05	SFE0.1	SFE0.2	*p* Value
LZM (μg/mL)	3.89 ± 0.26 ^a^	4.00 ± 0.25 ^b^	4.17 ± 0.21 ^c^	4.02 ± 0.29 ^b^	0.027
Complement C3 (g/L)	3.70 ± 0.09	3.70 ± 0.07	3.71 ± 0.09	3.69 ± 0.08	0.978
IgM (μg/mL)	54.60 ± 7.79	63.81 ± 10.88	64.25 ± 11.33	61.65 ± 16.91	0.200

Values (mean ± S.D.) (*n* = 6) in the same line with different superscript letters were significantly different from each other (*p* < 0.05). LZM, lysozyme; IgM, immunoglobulin M.

## Data Availability

Data is contained within the article.

## Abbreviations

IBW, initial body weight; FBW, final body weight; WGR, Weight gain rate; SGR, Specific growth rate; FR, Feeding rate; FCR, Feed conversion ratio; SR, Survival rate; PER, Protein efficiency ratio; HIS, Hepatosomatic index; VSI, Viscerosomatic index; CF, Condition factor; NF-κB p65, nuclear factor kappa B–p65; IκBα, inhibitor of κBα; TNF-α, tumor necrosis factor-alpha; IL-1β, interleukin-1 beta; TGF-β, transforming growth factor beta; IL-10, interleukin -10; Nrf2, nuclear factor erythroid 2-related factor 2; Keap1, kelch-like ECH-associated protein 1; Trx2, thioredoxin 2; TrxR2, thioredoxin reductase 2; GPx, glutathione peroxidase; GR, glutathione reductase; GST, glutathione S-transferase; SOD, superoxide dismutase; CAT, catalase; RPS4, ribosomal protein S4.
